# Sharing our excitement for structural science through mentorship

**DOI:** 10.1063/4.0000285

**Published:** 2025-02-10

**Authors:** Gerald F. Audette

**Affiliations:** Department of Chemistry & The Centre for Research on Biomolecular Interactions, York University, 4700 Keele St., Toronto, Ontario M3J 1P3, Canada

## Abstract

One of the most important means by which we can share our enthusiasm for structural science is our mentorship of trainees. Our trainees at all levels gain more than just technical skills from the time we spend with them; they develop their own appreciation and excitement for structural science that they then can spread through their connections and contacts. We play an important role, through our mentorship, in encouraging that excitement, fostering inquiry, and passing on that excitement to others. We often recount where our enthusiasm began, with one or more professors, mentors and/or colleagues whose excitement was infectious and helped us along our own professional journey and development of our own mentorship philosophies. In the current article, I outline how several mentors, including Professors Michael James, Louis Delbaere, Wilson Quail, and others, instilled that excitement for structural science in me and provided examples from which I have developed my perspective on mentorship and how we can pay it forward, supporting and instilling excitement in our trainees.

As part of the 2024 ACA Transactions Symposium, *Structural Science: Sharing Excitement & Appreciation*, I was invited by the organizers to share some thoughts on Michael James (1940–2023), who passed away in the autumn of 2023. Knowing that a scientific obituary for Michael has been published in *Acta Crystallographica*,^1^ and a more reflective remembrance is in the *ACA RefleXions* newsletter,^2^ I sought to look at how my mentors, including Michael James, Louis Delbaere, Wilson Quail, and others, shaped my views on how we share our excitement for structural science in the most direct manner, through the mentorship of our trainees.

How do we foster excitement in the structural sciences in our students and develop our own approach to mentorship? We all develop our engagement in structural science and approaches mentoring from our own experiences and mentors along our professional journeys. When I reflect on my experiences with mentors and colleagues along my professional path, I find that the key aspects of mentorship can be summarized as follows:

1. Academic and Research Opportunities
2. Enthusiasm and Support
3. Scientific Rigour and Building Independence
4. Fostering Community
5. Paying it Forward

I recognize that one's own philosophy toward mentorship is influenced by their own experiences; however, many successful mentors share components of the above in greater or lesser amounts as they develop their mentorship style. I outline below my path in developing that excitement for structural science, how it has shaped my perspective on mentorship, and how I look to share that excitement with my trainees as they embark on their scientific and professional careers.

## EARLY INFLUENCES AND SCIENTIFIC BEGINNINGS

Do we remember when we got excited by structural science? Or even more broadly, what got us excited about science in general,? It all starts somewhere, is different for everyone, and is often fondly remembered when we look back on that moment. For Michael James, according to his obituary,^1^ it was an eventful experiment with liquid oxygen in his basement while growing up in Winnipeg, Manitoba, Canada. For me, while it was less of a bang, it was still an impactful foray into experimental science. I recall a science project in elementary school where I tested blood groups in an experiment akin to what Karl Landsteiner engaged in around the turn of the 20th century to discover the ABO(H) blood groups. I am not sure how we got past health and safety regulations at the Sturgeon General Hospital in St. Albert, Alberta (just north of Edmonton), but with a mother who was a nurse at the hospital (my father is a chemist), somehow there I was mixing blood samples in test tubes. *Voila, a love for inquiry was born*. I was struck by how we can take a straightforward hypothesis, such as “what would happen if I mixed these different blood samples together?,” through the observable results of that experiment and develop a deeper understanding of the workings of the world around us. I continue to ask straightforward questions about the world around us; I have found that straightforward questions to complex problems are both more approachable experimentally and more readily understood by non-experts, trainees, and colleagues.

Many of us have similar stories of early interest in science and inquiry fostered by parents, teachers, etc. that brought us to that point of initial excitement in discovery. While the above explains how I got that spark of interest in science and inquiry in general, what was the trigger for a lasting engagement in structural science? Well, that came when I began my undergraduate years at the University of Alberta, interacting with Michael James and others on those first steps of my academic and professional path.

## ACADEMIC INFLUENCES, RESEARCH OPPORTUNITIES, AND DEVELOPMENT OF MENTORING PHILOSOPHY

**FIG. 1.**
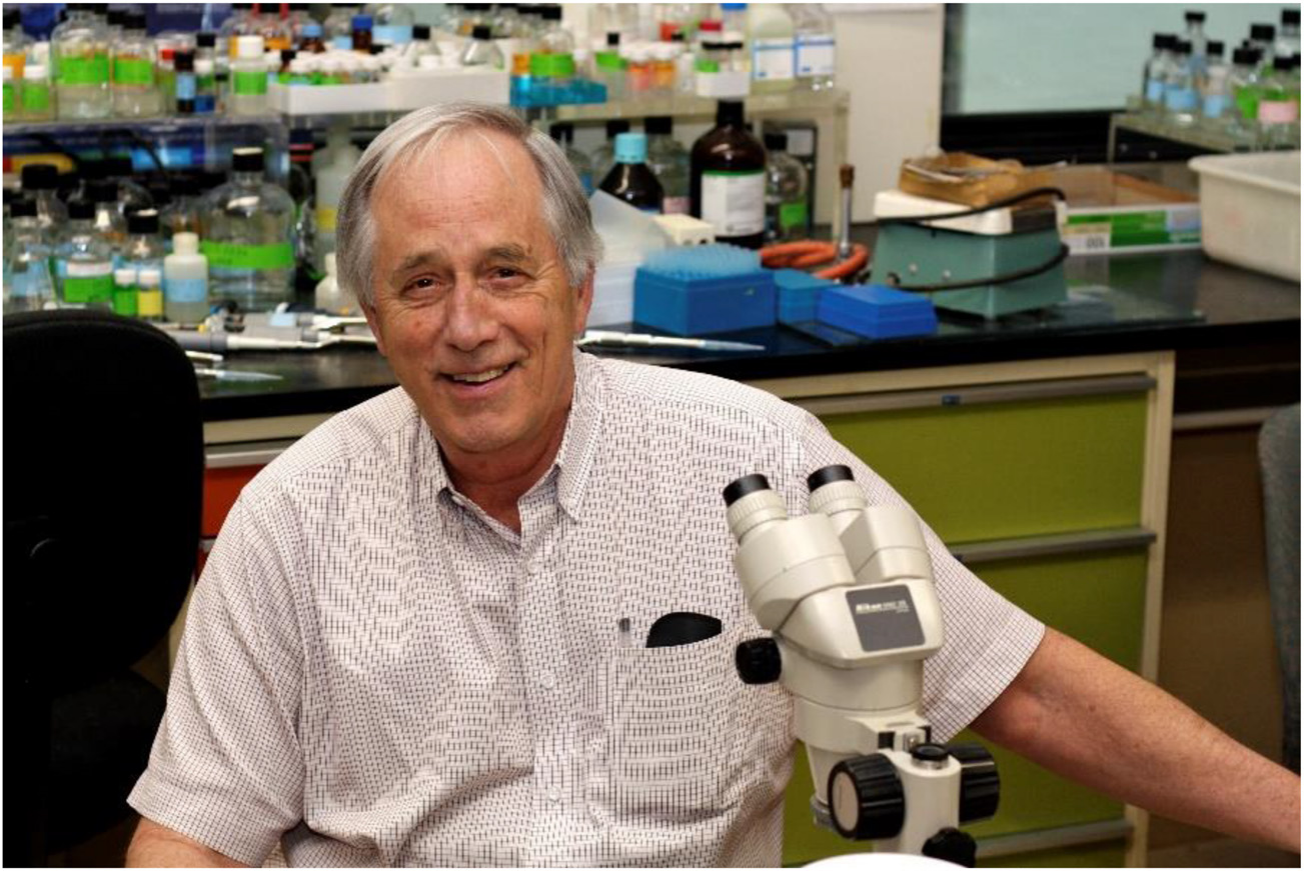
Professor Michael James, a pioneer in macromolecular crystallography in Canada, in the lab in 2010. Michael joined the Department of Biochemistry at the University of Alberta in 1968 and spent his career exploring protein structure and function and mentoring many crystallographers and structural biologists. Reproduced with permission from Glover *et al. Acta Cryst* **D79**, 953–955 (2023).^1^ Copyright 2024 International Union of Crystallography.

**FIG. 2.**
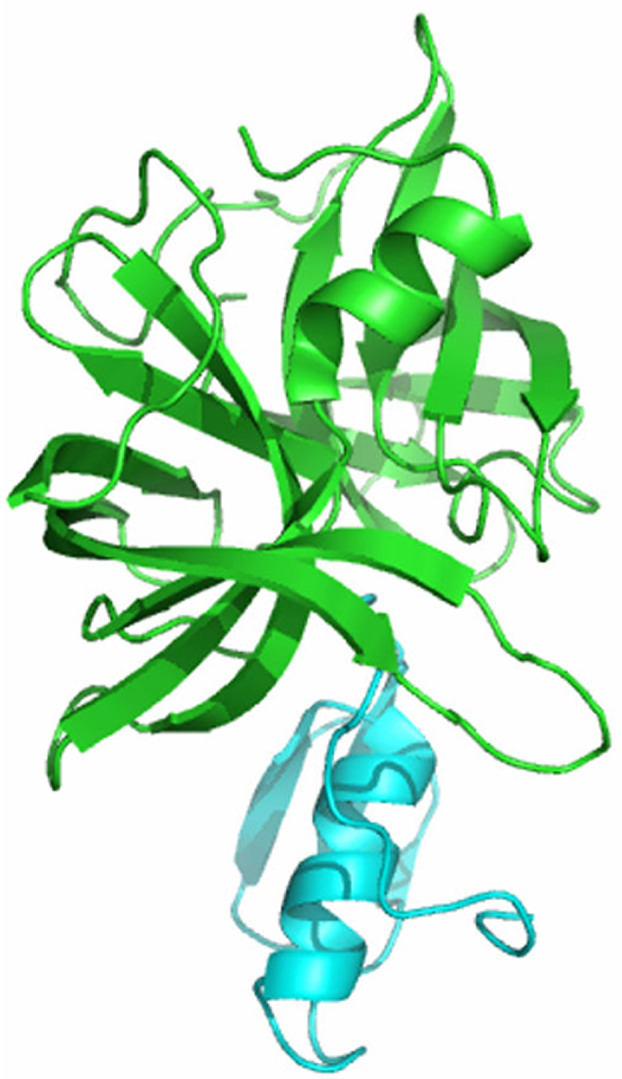
The first three-dimensional protein structure determined in Canada, *S. griseus* protease B, was solved by Louis Delbaere and Michael James in 1974.^3^ Shown is the re-refined structure of Read *et al.* of the protease (green) complexed with the third domain of the ovomucoid inhibitor (blue) reported in 1983 (PDB ID 3SGB).^4^ Image generated in Pymol.^10^

Dr. Michael James (Fig. 1) is, quite rightly, a founder of macromolecular crystallography in Canada. Michael completed his DPhil with Dorothy Hodgkin in 1966, following which he engaged in postdoctoral research in the Department of Chemistry at the University of Alberta. In 1968, Michael joined the faculty Department of Biochemistry at the University of Alberta and became the first protein crystallographer in Canada. It was here he spent his 50-plus year career focusing on the structure and function of proteolytic enzymes and other medically relevant proteins, mentoring trainees of all levels, and sharing his excitement for structural science. Michael's group was the first to determine the three-dimensional structure of a protein in Canada, the *Streptomyces griseus* protease B (Fig. 2), research led by then postdoc Louis T.J. Delbaere (1943–2009).^3^ Michael was a founding member of the highly renowned Medical Research Council (MRC; now the Canadian Institutes for Health Research) Group in Protein Structure & Function in 1974.^1^ The MRC group, which included Profs. Cyril Kay, Larry Smillie, Brian Skyes, Robert Hodges, Robert Fletterick, Charles Holmes, Wayne Anderson, and Zygmunt Derewenda, operated between 1974 and 2000, published over 1600 papers, and trained more than 250 students and postdoctoral fellows. If one was in Western Canada and interested in structural science, it was hard to avoid the influence, direct or indirect, of Michael and the MRC group.

It was into this environment that I entered my undergraduate years at the University of Alberta, studying biochemistry in the early 1990s. Little did I appreciate at the time the strength of the University of Alberta in protein biochemistry and structural science, though I am most certainly grateful for it. We tend to link our first spark of excitement into a specific scientific discipline to our undergraduate years, and I was no exception. Early lectures in protein chemistry with Prof. Cyril Kay certainly piqued my interest in proteins vs carbohydrates or lipids. However, it was a pair of courses on Protein Structure and Function, partially taught by Michael, and Physical Biochemistry, partially taught by Zygmunt Derewenda, that sealed the deal for me. I was fascinated by Michael's passion and his explanations of the serine proteases, using the *S. griseus* protease B as an example, as to how structure and function are linked; I wanted to understand how we could make those characterizations to contribute to our understanding to biochemical processes. The course on physical biochemistry had sections on analytical ultracentrifugation and protein hydrodynamics, NMR, and x-ray crystallography. I loved it (my friends thought I was crazy), and I knew this was what I wanted to study. Now how to do that? I, like so many of our students, had to pluck up the courage and approach one of my professors to see if they had let me into their lab and try my hand at some “real research.” Unfortunately, neither Michael nor Zygmunt had any more open spaces for a summer research student that year. However, Michael had seen me hanging about the department (I had a part-time job in the departmental stores) and said I could come in from time to time and set up a crystallization plate or two. He introduced me to his long-time technician, Maia Cherney, and I got to setting up crystal plates! It did not matter what the protein was, probably lysozyme, but I got a bit of hands-on lab experience. That is the first thing I took away and have tried to emulate—look to give research opportunities to students. Even a student lab volunteer coming in part-time doing something small and maybe not targeted to a specific project (though that really does help) offers them something tangible—a lab experience—and less tangible, being part of a group and contributing.

As my undergraduate years neared their end, I started looking into graduate studies in protein crystallography. When I announced to my family that this was what I wanted to do with my life, my father Robert started chuckling. I was confused by this reaction, and oddly annoyed, until he explained that he himself had done some crystallographic analyses of several ferrate compounds^5^ while completing his PhD in Chemistry with J. Wilson Quail (1936–2023) at the University of Saskatchewan (U of S), in Saskatoon, Saskatchewan (now home to the Canadian Light Source), back in the late 1960s to early 1970s. Both my parents were very supportive of my pursuit of structural science, especially after Robert stopped chuckling as to how things can repeat themselves. After multiple applications being sent out, I secured a graduate position with Louis Delbaere (1943–2009)^6,7^ and Wilson Quail at the University of Saskatchewan. This became the second important influence on my thoughts surrounding academic opportunities; look to provide academic opportunities to not just the top students but also to that enthusiastic student who might not be all “straight A^+^.” I was told by an undergraduate faculty advisor that my grades were strong enough to get into graduate school but not quite there for scholarships/funding and so not to expect much. Louis and Wilson interviewed me, looked past the scholarship/funding issue (I worked as a teaching assistant throughout graduate school), and took a chance on an enthusiastic young scientist. By keeping a broader view on a student's academic progress, we can look to identify those who have a clear enthusiasm for research and structural science. That vote of early confidence by a supervisor taking on a graduate student can result in increased perseverance and growth of that younger scientist in the early stages of their career.

I also gained another valuable insight into mentorship from the process of finding a graduate position; be enthusiastic and supportive of a young scientist's journey, even if it is not with you. During a summer research project with Brian Sykes doing protein NMR, I recall when I mentioned to Michael and Brian that I was going to pursue graduate work in crystallography with Louis. Michael was enthusiastic about my pursuit of crystallography and not NMR, a point for which he needled Brian in a good-natured fashion, and for which they both got a laugh out of. That I was not pursing graduate studies with either of them was less important than enthusiastically supporting a young scientist on their academic and research journey. What was also apparent from this interaction was that in the structural sciences, there are many methods that we can use to gain structural information, each with their own strengths and technical challenges. Different methods are not at odds with one another but rather are complementary approaches; by asking questions from different viewpoints/methods, we gain a more comprehensive understanding the world around us. We should be open to engaging in multiple methods/techniques to answer research questions. This supportive interaction from faculty mentors certainly made an impression, both in terms of my approach to research as a structural scientist and to my mentoring philosophy.

**FIG. 3.**
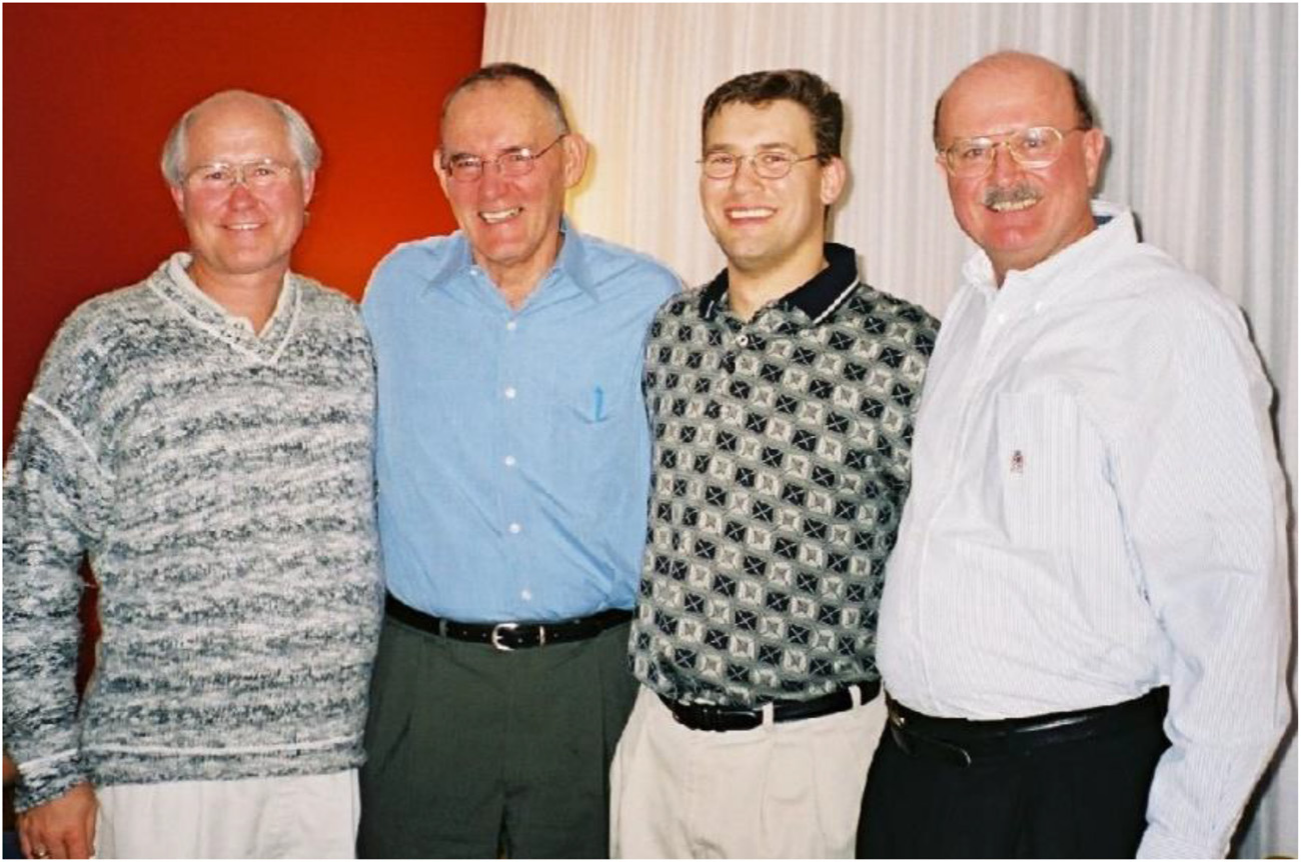
Structural science on the Canadian Prairies. Taken at a group gathering to celebrate the completion of my PhD in autumn 2001, left-to-right are Robert Audette, Wilson Quail, Gerald F. Audette, and Louis Delbaere. It was a bit of an inside joke amongst us that Robert was Wilson's first PhD student (back in the 1960s), I was his last (1995–2001), and that we “bookended” his career at the University of Saskatchewan; Wilson retired in 2003. Getting together to celebrate and fostering a deep sense of community in our group was critical to Louis and Wilson; it is something I look to emulate with my research group and trainees. Reproduced with permission from the personal archives of G.F. Audette. Copyright G.F. Audette.

Louis Delbaere joined the Department of Biochemistry at the University of Saskatchewan in 1979, following his postdoctoral work with Michael at the University of Alberta. It was there he met Wilson, and the two colleagues began a long and fruitful collaboration in structural science, sharing facilities and co-supervising many students. I started on a project that was thought to be straightforward, a quick soaking of a blood-group specific carbohydrate into crystals of a lectin (the first lectin from *Ulex europeaeus*; UE-I) and determine the crystal structure of the complex. While it turned out to be much more involved, it was oddly serendipitous given my early experimental foray with blood groups for that school science project years before. Louis and Wilson were always steadfastly supportive, encouraging, and pushing for my growth as a structural scientist. I was encouraged to explore multiple approaches to unravel the specifics of the lectin–carbohydrate interaction, including mass spectrometry, NMR, various spectroscopic methods, etc., even if the main goal was the determination of a crystallographic structure. Thinking more recently, this could also include Cryo-EM, SAXS, multi-angle light scattering, molecular dynamics, and other methods, and is something that makes a more well-rounded structural scientist. Many of our projects rely on multiple analytical techniques, and a modern structural scientist should explore appropriate methods when asking our research questions; projects can be tricky by one method yet more amenable by another. Supporting the engagement of multiple techniques in research not only facilitates a robust understanding of the system one is studying, but it also fosters independence, self-confidence, and a willingness to expand our trainees' knowledge base, which are critical for their growth and future careers. We eventually determined the crystal structures of two carbohydrate complexes of the UE-I lectin,^8,9^ and I defended my PhD (Fig. 3). Throughout my studies, the support, enthusiasm, and encouragement of my mentors, their dedication to scientific rigor rather than the flash of a story, and to following the data (both Louis and Wilson always said “the electron density does not lie, trust it”), have been critical to my approach in (hopefully) fostering the same in my research group.

## FOSTERING COMMUNITY

Another important aspect of mentoring and sharing our excitement for structural science is looking to foster community. I recall during my summer studentship with Brian Sykes' group that there were often morning coffee breaks and at least one lab-wide gathering outside the lab. This allowed for a more relaxed engagement with all members of the group. In addition, throughout my time in graduate studies with Louis and Wilson, they both made significant efforts to foster a sense of community within the group, even if it was not a regular coffee time. Louis and Carol, along with Wilson and Florence, were always welcoming the group into their homes for seasonal gatherings and dinners to celebrate the lab members' achievements, student defenses, etc. These gatherings let us talk about things other than our immediate research triumphs or tribulations, and let the group gather with our significant others to be together. I certainly found those gatherings meaningful moments, and reflection on them crystallizes the importance of community in my perspective of mentorship. The more we see our groups as a supportive community rather than a competitive one, the more we can rely on them in a positive way when things are difficult. All research projects have challenges; knowing you have a supportive group to rely on, bounce ideas off of, lend a hand or brainpower to, and to celebrate those breakthrough moments with is important to our perseverance through tricky projects and hours of lab work, data collection/processing, model building and refinement, etc.

We are often first exposed to the wider scientific community during our graduate studies, though there are certainly opportunities for welcoming enthusiastic undergraduates to present at the annual ACA meetings. On my career path, Louis and Wilson felt, as I certainly do, that attending scientific meetings is critical for a trainee's scientific growth and the development of their own professional networks. This was mainly through ACA meetings, and my first foray into the broader structural science community was the 1996 ACA/IUCr meeting in Seattle, WA, USA. Louis and Wilson were always there giving introductions to colleagues and encouraging members of their groups to fully engage in the community. They encouraged us, and led by example, to explore all the posters at a meeting, not just the ones we were familiar with, to chat with the presenters about their projects, and to ask questions of speakers if we found something interesting. The structural science community is very welcoming, but it can be daunting for trainees to step out and say “Hi” to the people in the field that they read of in papers. By helping make connections, and encouraging their engagement broadly, we bring our trainees more into the community and help them establish their own growing professional networks. By visiting those posters at the back of the hall, talking with the presenters, and sharing enthusiasm for their research, we demonstrate an excitement for structural science and a welcoming air that fosters future interactions and engagement.

We should also remember that trainees, when they move on to other projects, labs, etc. pay attention to our engagement, on-going or otherwise, with them. I recall that throughout my graduate studies, Michael James always seemed to be aware of my progress whenever we met at ACA meetings. Given that my main research project focused on blood group recognition and was a collaborative effort with another giant of Canadian Chemistry, Raymond Lemieux (1920–2000), Michael was, unbeknownst to me at the time, well versed in the system. Michael, Ray, and Louis were long-time colleagues, with Louis having been a postdoc with Ray before joining Michael's group. Michael seemed to be intimately aware of my project, which was surprising to me at the time, but less so now. Michael was just that interested in the science that he kept abreast of these things. However, it certainly made an impression to me on how to retain engagement and pass on our excitement to the next generation by being aware of and enthusiastic about their on-going research or career progress.

## PAYING IT FORWARD

**FIG. 4.**
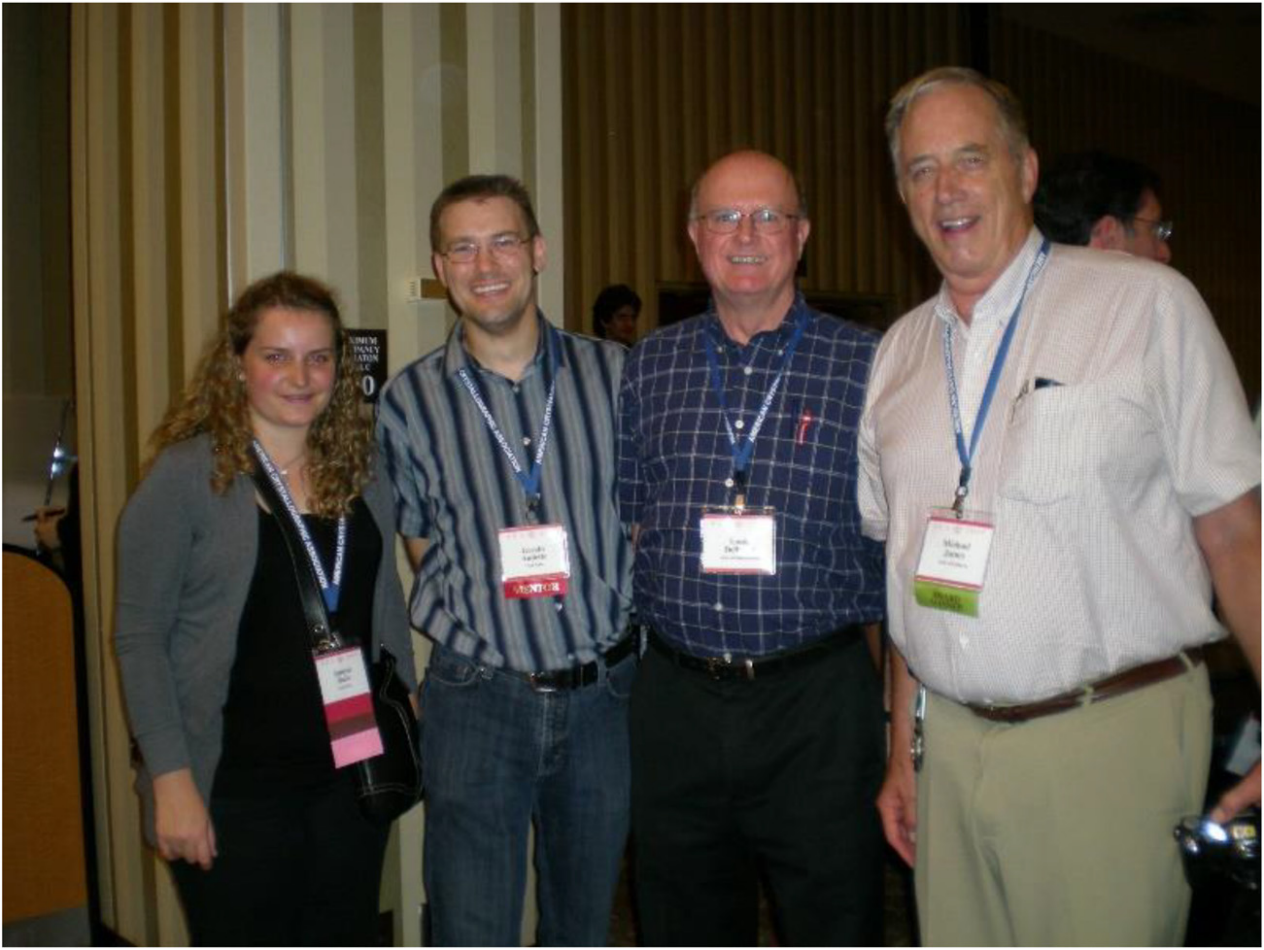
Four generations of Canadian structural scientists. Taken at the 2009 ACA meeting in Toronto, left-to-right are Agnesa Shala-Lawrence, Gerald F. Audette, Louis Delbaere, and Michael James. Michael and Louis' guidance, mentorship, and evident passion and excitement for structural science are things that have guided me in how I approach my training and mentoring. I look to pass my excitement for structural science forward to the next generation through engagement, questions, and encouragement. Their successes are our success. Reproduced with permission from the personal archives of G.F. Audette. Copyright G.F. Audette.

When one embarks on their independent career, we are now able to pay it forward, and the supports we give and receive help shape our own research group's trajectory. I joined the Department of Chemistry at York University in Toronto in 2006, following a postdoc at the University of Alberta with Bart Hazes and Laura Frost, and started building my research group. I have been fortunate that many colleagues have been supportive and collegial throughout, and I look to return that support when I can. Michael, Louis, and Wilson were all extremely supportive, offering advice and insights when asked, and being engaged and interested in my trainees' research when we met at conferences. The 2009 ACA meeting in Toronto stands out in my mind, both as the last meeting for Louis, who passed away later that year, but also as one where both Louis and Michael fully engaged with my then relatively new PhD student, Agnesa Shala-Lawrence, a first-time ACA attendee. Both Louis and Michael made it a point to visit Agnesa's poster, ask pertinent and probing questions, impart sage advice, and offer heartfelt encouragement (Fig. 4). It was, in my mind, the embodiment of how we should be fostering that excitement for structural science; through a genuine engagement and enthusiasm for the research and development of our trainees, especially if they are experiencing challenges in their projects (and all projects have challenges). These interactions may not seem like much to us when we are asking questions at a poster session. However, they certainly resonate with our trainees and are the building blocks of their confidence, independence, and development of their own professional networks. We are paying it forward, and it makes a lasting positive impression.

## CONCLUDING THOUGHTS

Mentorship of our trainees is a main route to foster excitement in the structural sciences. We develop our approach to mentorship through experiences with our mentors and colleagues along our career path and adapt them to our own circumstances. From my perspective, the opportunities and mentorship I have had from many individuals have shaped my approach to mentorship and engagement in structural science. From initial beginnings that foster a student's interest in discovery, we should provide academic and research opportunities to get them engaged. By understanding that not all students take the same career path, we can provide opportunities to many students, find those that really shine, and provide a lasting impact on their professional careers, wherever their journey takes them. We need to be enthusiastic about our research and its impact, ask straightforward questions, and not be afraid to tackle difficult problems. Challenging projects are often the most engaging, even if they take time to see positive results, which can be tough for a student who wants an immediate result but is struggling with a tricky problem. Being genuinely engaged, encouraging, and supportive of them will keep them engaged, working hard, and help them build resilience and self-confidence. Next, we need to be rigorous in our approach to our science and look to foster independence, self-confidence, and a willingness to explore different approaches to answering our research questions in our trainees. If we expect the best from ourselves, so will our trainees. We also need to foster community and remember to celebrate. We all work hard on our own research and projects, so gathering outside the lab, even if it is just on-campus for coffee or tea regularly, builds teamwork, a positive sense of community, and lasting memories and friendships. Finally, we need to pay it forward, through genuine and enthusiastic support for trainees, both ours and others. They will then carry that excitement and commitment to structural science as they move on to their own careers.

I am grateful to my mentors, including Louis, Wilson, Michael, Bart, Laura, and many others, for their support and encouragement, and to my students for their trust and enthusiasm to tackle challenging projects as they work toward their career goals. I of course must thank the ACA and my colleagues for being so passionate about structural science, lasting friendships, excellent discussions, insightful critiques, and encouragements of our research. It is through these interactions that we build our community, foster excitement in structural science, and pay it forward.

## Data Availability

Data sharing is not applicable to this article as no new data were created or analyzed in this study.
